# The non-canonical CDK2 activator SPY1 uses separable mechanisms to activate phosphorylated and unphosphorylated CDK2

**DOI:** 10.1042/BCJ20260441

**Published:** 2026-09-10

**Authors:** Liam P. Fawcett, Nicholas M. Levinson

**Affiliations:** 1 Department of Pharmacology, University of Minnesota, Minneapolis, MN 55455, U.S.A.; 2 Masonic Cancer Center, University of Minnesota, Minneapolis, MN 55455, U.S.A.

**Keywords:** CDK2, Cooperativity, cyclin dependent kinases, RINGO, SPEEDY, SPY1

## Abstract

The cyclin-dependent kinases (CDKs) regulate cell cycle progression subject to strict control by activating cyclin subunits and by site-specific phosphorylation on the activation loop. These mechanisms are highly conserved across eukaryotic organisms. The non-canonical CDK activator Speedy A (SPY1) is unrelated in sequence to the cyclins and activates CDK2 in G1/S phase in somatic cells and promotes meiosis in germ cells without requiring activation-loop phosphorylation of CDK2 on T160. This has been attributed to a single aspartic acid residue of SPY1 (D136) that mimics T160 phosphorylation by forming similar salt-bridging interactions that stabilize the active conformation of the SPY1–CDK2 complex. Here, we show that SPY1 activates both phosphorylated and unphosphorylated CDK2 with similar efficacy to its canonical activator CyclinA. FRET experiments show that SPY1 binding causes a similar magnitude of CDK2 conformational shift to that triggered by CyclinA binding to phosphorylated CDK2, but regardless of the kinase phosphorylation state. SPY1 binding allosterically enhances binding of ATP-competitive CDK2 inhibitors to a similar degree as CyclinA, resulting in similarly high-affinity inhibitor binding and inhibition. Remarkably, we found that the D136A mutant of SPY1, while inactive towards unphosphorylated CDK2, can still activate phosphorylated CDK2 as effectively as WT SPY1, demonstrating functional non-equivalence of its two activation mechanisms.

## Introduction

Tightly regulated cell growth and proliferation are fundamental necessities of multicellular life. In eukaryotic cells, progression of the cell cycle is tightly regulated by the cyclin-dependent kinases (CDKs) [1]. The core cell cycle CDKs (CDK1, CDK2, CDK4, and CDK6) are inactive in their monomeric form, and their activation is governed by a coordinated set of regulatory mechanisms. Two conserved mechanisms involve phosphorylation of a conserved threonine residue (T160 in CDK2) in the activation loop (A-loop) and binding of a regulatory cyclin subunit [2]. Activation loop phosphorylation is performed continuously in actively cycling cells by the CDK-activating kinase (CAK) complex [3]. In contrast, the expression of the cyclins fluctuates throughout the cell cycle in a precisely orchestrated manner that serves to activate particular CDKs at the appropriate time to faithfully execute each phase of the cell cycle, with CyclinD–CDK4 controlling cell cycle entry, CyclinE–CDK2 S-phase entry, CyclinA–CDK2 S-phase completion, and CyclinB–CDK1 M-phase entry. An additional level of regulation is mediated by the CDK inhibitor proteins of the p21/p27 family, which block several cell cycle CDKs, and the INK4 family, which inhibits CDK4/6 [4,5].

The catalytic activity of CDKs depends on the precise positioning of key structural elements within the kinase domain, including the A-loop and αC-helix [6]. Monomeric CDK2 adopts an auto-inhibited conformation in which the αC-helix is swung out of the active site, breaking a conserved Glu-Lys salt bridge required for catalytic activity (“αC-out”), while the A-loop is folded up towards the rest of the kinase domain, blocking substrate binding (“A-loop_in_”) [7]. Binding of CyclinA triggers a conformational rearrangement of these elements, where the αC-helix rotates inwards towards the active site (αC-in) and the A-loop folds outwards (A-loop_out_) [8]. In the unphosphorylated form, this cyclin–CDK2 complex is inactive because the A-loop is misaligned and cannot support the binding of peptide substrates. To form the active cyclin–CDK2 complex, the A-loop must also be phosphorylated on T160. The T160 phosphothreonine side chain becomes partially buried in the binding interface between the cyclin and the CDK and forms salt bridges with conserved arginine residues in CDK2 that pin the A-loop in an extended conformation in which it forms a platform for the binding of substrates [9]. While the T160 residue is rapidly phosphorylated and dephosphorylated in monomeric CDKs by the action of the CAK and KAP phosphatase, respectively, cyclin subunit binding can block dephosphorylation, unleashing stable and active CDK–cyclin complexes to complete cell cycle progression [10]. Crystal structures of numerous CDK–cyclin complexes have shown that the stabilizing interactions nucleated by the A-loop phosphothreonine residue are a highly conserved feature of the CDK family [11–15].

Speedy (SPY1) is a non-canonical CDK activator first identified in Xenopus for its role in oocyte maturation and M-phase entry [16,17]. Five mammalian paralogues are known (Speedy A–E) that are predominantly expressed in germ cells, where they play a central role in meiosis [18,19]. However, Speedy A (hereafter referred to as SPY1) is also expressed in somatic cells and has been shown to contribute to the G1/S transition in a manner that is dependent on CDK2 [20]. Importantly, SPY1 is capable of bypassing several key features of canonical CDK/cyclin regulation, including inhibition by p21 and, most notably, the requirement for T160 A-loop phosphorylation of CDK2 [21]. The crystal structure of unphosphorylated CDK2 bound to SPY1 showed that SPY1 causes a conformational change of CDK2 that closely resembles that triggered by cyclin binding, with the αC-helix rotating inwards and the A-loop folding outwards into the A-loop_out_ state [22]. However, in the SPY1–CDK2 complex the A-loop is correctly positioned for substrate binding despite the absence of phosphorylation on the T160 residue [22]. The ability to induce this fully active conformation of the A-loop is attributed to a stretch of acidic amino acids in SPY1 (residues 134–139) that form electrostatic interactions at the interface between SPY1 and the A-loop of CDK2. Within this conserved “acidic loop”, residue D136 takes the place of the T160 phosphothreonine side chain and acts as a phosphomimetic residue by forming a similar network of salt-bridging interactions with the CDK2 arginine residues as observed in canonical cyclin–pCDK complexes. Sequence analysis of SPY1 orthologs has shown that the acidic stretch of amino acids is conserved across vertebrate species, indicating a universal mechanism for SPY1 activation of CDKs [18].

Previous studies of the SPY1–CDK2 complex were hampered by the instability of isolated SPY1 protein, which interfered with biochemical and biophysical characterization of its binding interaction with CDK2. Here, we identified a SPY1 construct that can be stably isolated in pure form and used biophysical and biochemical methods to characterize the binding interaction between SPY1 and CDK2 and probe the conformational shift towards the CDK2 active state triggered by SPY1 binding. We found that SPY1 binds to unphosphorylated CDK2 with similarly high affinity as CyclinA and potently activates the kinase. We show that ATP-competitive CDK2 inhibitors bind with surprisingly similar affinities to the SPY1–CDK2 and CyclinA–CDK2 complexes. Using FRET experiments tracking CDK2 conformation changes, we show that the allosteric coupling between CDK2 and SPY1 is remarkably similar to that of CyclinA despite the striking difference in their regulatory mechanisms. Finally, we show that mutation of the SPY1 D136 residue prevents activation of unphosphorylated CDK2 by SPY1 but leaves activation of phosphorylated CDK2 intact, essentially converting SPY1 into a cyclin-like activator protein.

## Results

### SPY1 potently activates CDK2 independently of A-loop phosphorylation by triggering a conformational shift to the active state

**Figure 1:**
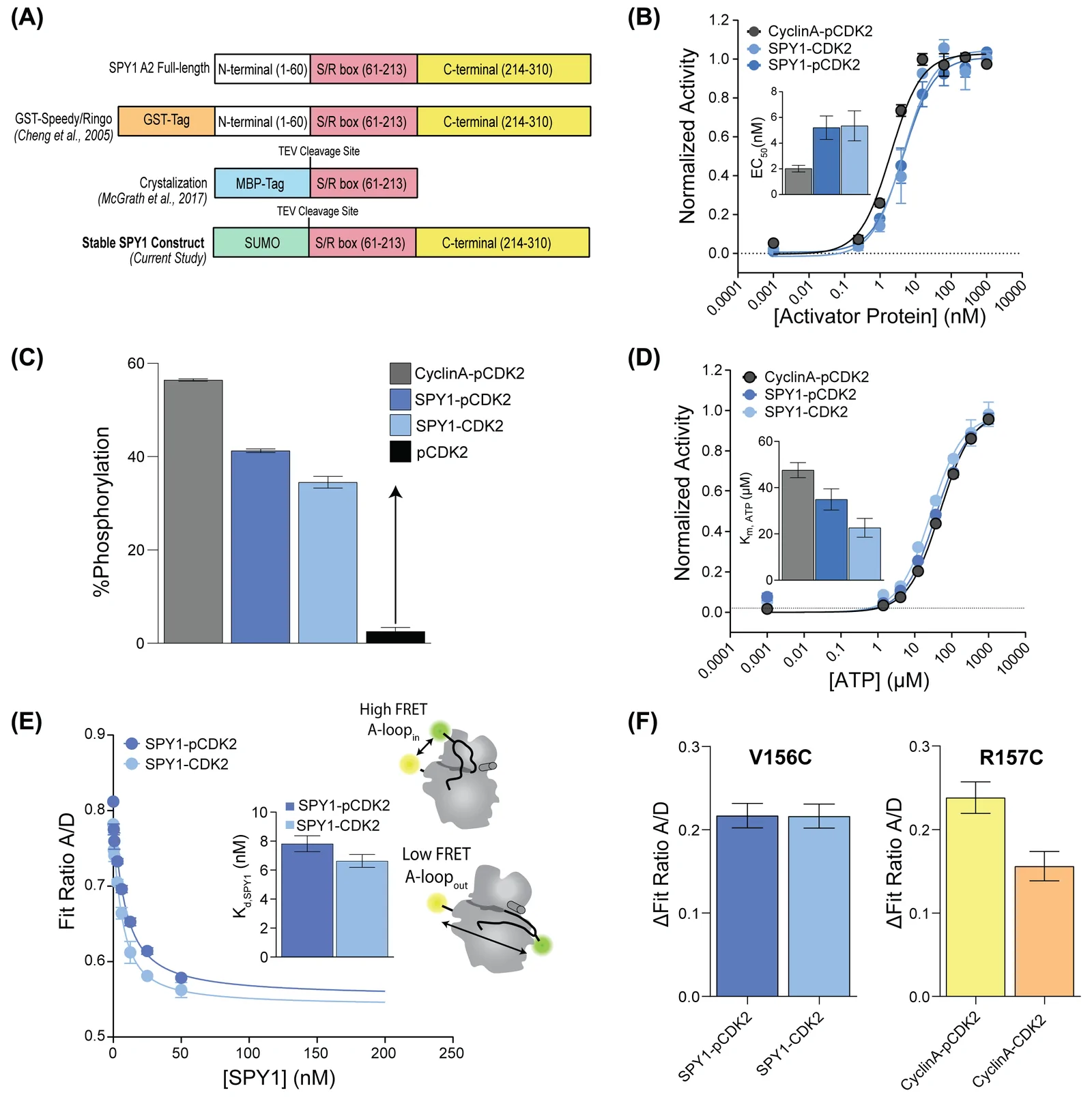
SPY1 potently activates CDK2 independently of A-loop phosphorylation by triggering a conformational shift to the active state (**A**) Schematic representation of the SPY1 construct (bottom) used in the present study compared with full-length human SPY1 (top) and constructs used in previous biochemical studies and structure determination (middle). (**B**) Activity assays performed with SPY1 and pCDK2 (dark blue) or SPY1 and CDK2 (light blue) compared with CyclinA and pCDK2 (gray) at different concentrations of the activator proteins. Data were normalized to the maximal activity of each complex. The inset shows the parameters derived from fitting the primary data. (**C**) Maximal unnormalized activity measured at saturating activator protein for CyclinA–pCDK2 (gray), SPY1^WT^–pCDK2 (dark blue), SPY1^WT^–CDK2 (light blue), and monomeric pCDK2 (black). Data represent mean ± SEM from a single representative experiment with *n* = 3 replicates. (**D**) Activity assays performed with different ATP concentrations and saturating activator protein concentrations for CyclinA–pCDK2 (black), SPY1^WT^–pCDK2 (dark blue), and SPY1^WT^–CDK2 (light blue). Data were normalized to the maximal activity for each complex. Inset shows K_m,ATP_ values derived from fitting the data in the main panel. (**E**) Ratiometric FRET measurements for SPY1 titrations onto pCDK2 (dark blue) and CDK2 (light blue) using the CDK2^V156C^ FRET sensor. Data were fit to the quadratic 1:1 binding model (see Methods). The inset shows K_d_ values derived from these fits. (**F**) Total change in FRET signal due to binding of SPY1 (left) or CyclinA (right) to CDK2 or pCDK2. Plotted data represent mean ± SEM; *n* = 3 independent experiments.

Previous studies of SPY1 utilized truncated constructs containing only the central Speedy/Ringo (S/R) box region required for CDK activation (residues 61–213) and were impeded by the instability of the resulting protein that necessitated co-purification with CDK2 (Figure 1A) [22,23]. We extended the expression construct to include the 96 amino acids C-terminal to the S/R box (residues 61–310) and added a TEV-cleavable N-terminal SUMO solubility tag (Stable SPY1 Construct, Figure 1A). The N-terminal segment of SPY1, residues 1–60, was not included, as it is dispensable for CDK2 activation [24]. Using this construct, we were able to purify stable, isolated SPY1 protein to characterize its interaction with CDK2. CDK2 was prepared with (“pCDK2”) and without (“CDK2”) phosphorylation on T160 via co-expression with yeast CAK (see Methods).

Our SPY1 construct strongly activated CDK2 in kinase activity assays that utilize a fluorescently labeled substrate peptide with a consensus CDK recognition sequence, including an arginine residue at the P + 3 position known to enhance recognition by CyclinA–pCDK2 (see Methods) [25]. Assays performed at different concentrations of the activator protein (SPY1 or CyclinA) revealed that SPY1 has an EC_50_ value of 4.5 ± 0.8 nM for CDK2, with no significant difference between phosphorylated and unphosphorylated CDK2 (Figure 1B). This is similar to the value of 2 nM measured for CyclinA activating pCDK2, as well as those reported for other cyclin–CDK complexes [26], suggesting that SPY1 evolved under selective pressure to compete with the high-affinity binding of the cyclins. When compared at saturating concentrations of their respective activator proteins, the SPY1–CDK2 complexes exhibited somewhat lower catalytic activity than CyclinA–pCDK2 (Figure 1C). To test if this difference might be due to different K_m,ATP_ values, we monitored activity as a function of ATP concentration. Fitting these data to a Michaelis-Menten model showed that the SPY1–CDK2 complexes have similar K_m,ATP_ values to the CyclinA–pCDK2 complex (Figure 1D). This suggests that the moderately lower activities observed for the SPY1–CDK2 complexes are due to different preferences for the substrate peptide used in our assays [23].

To measure the binding of SPY1 to CDK2 and track the ensuing conformational change, we designed a FRET sensor that monitors movements of the CDK2 A-loop in response to SPY1 binding. This sensor utilizes CDK2 protein labeled with donor and acceptor dyes on the comparatively stationary αD-helix (A93C) and the flexible A-loop (V156C), such that conformational changes of the A-loop lead to changes in FRET. This CDK2 sensor architecture is similar to one we previously used to study the effects of CyclinA binding [27], but with the V156C A-loop labeling site substituted for the R157C labeling site. This change was necessitated by the observation that the R157 residue forms an important salt-bridging interaction with the acidic loop of SPY1 [22]. Control experiments confirmed that high-affinity SPY1 binding and CDK2 activation are intact in the CDK2 V156C mutant (Figure 1E and Supplementary Figure S1G) but demonstrated that the mutation also weakens CyclinA binding. Consequently, in the FRET experiments described below, all SPY1 measurements were performed with the V156C version of the FRET sensor, whereas all CyclinA experiments were performed with the original R157C version. Note that experiments performed with both FRET sensors using a common set of CDK inhibitors confirmed that the two sensors provide very similar readouts of ligand-driven conformational change despite the different labeling sites.

We performed titrations of SPY1 protein using both T160-phosphorylated and unphosphorylated forms of the CDK2^V156C^ FRET sensor. The unphosphorylated form was prepared by treating the phosphorylated sensor with lambda phosphatase after dye labeling, yielding identical dye labeling patterns for both samples that permit direct comparison of their A/D ratios. In these ratiometric steady-state FRET experiments a reduction in the acceptor to donor (A/D) ratio indicates a shift from the high-FRET A-loop_in_ state to the low-FRET A-loop_out_ state (Figure 1E schematic). Addition of SPY1 led to pronounced decreases in the A/D ratio for phosphorylated and unphosphorylated CDK2^V156C^ that were nearly identical in magnitude (Figure 1F). These decreases in A/D ratio were also similar to those observed in control experiments with CyclinA binding to phosphorylated CDK2^R157C^, consistent with SPY1 triggering a similarly large conformational shift to the A-loop_out_ state (Figure 1F). However, in the case of CyclinA binding to CDK2, the large conformational shift requires phosphorylation on T160, and binding to unphosphorylated CDK2 yields a smaller conformational shift, reflected in a smaller reduction in A/D ratio, as reported previously [27]. This incomplete conformational shift is consistent with the inability of CyclinA to activate unphosphorylated CDK2. In contrast, the ability of SPY1 to trigger a similarly large conformational shift when binding to either phosphorylated or unphosphorylated CDK2 (Figure 1F) is consistent with its ability to strongly activate both forms of CDK2.

Fitting the SPY1 dose response curves measured by FRET yielded a value for K_d,SPY1_ (Figure 1E, inset) of 6.6 ± 0.89 nM for binding to unphosphorylated CDK2 and 7.8 ± 1.1 nM for binding to phosphorylated CDK2, in good agreement with the EC_50_ values measured above. These data confirm that SPY1 binds tightly to CDK2, that its binding is not substantially affected by phosphorylation on T160, and that in both cases it triggers a conformational shift that is similar in magnitude to that triggered by CyclinA binding to phosphorylated CDK2, consistent with full induction of the active A-loop_out_ state.

### CDK inhibitors show strong positive binding cooperativity with SPY1 and potent inhibition of SPY1–CDK2 complexes

**Figure 2:**
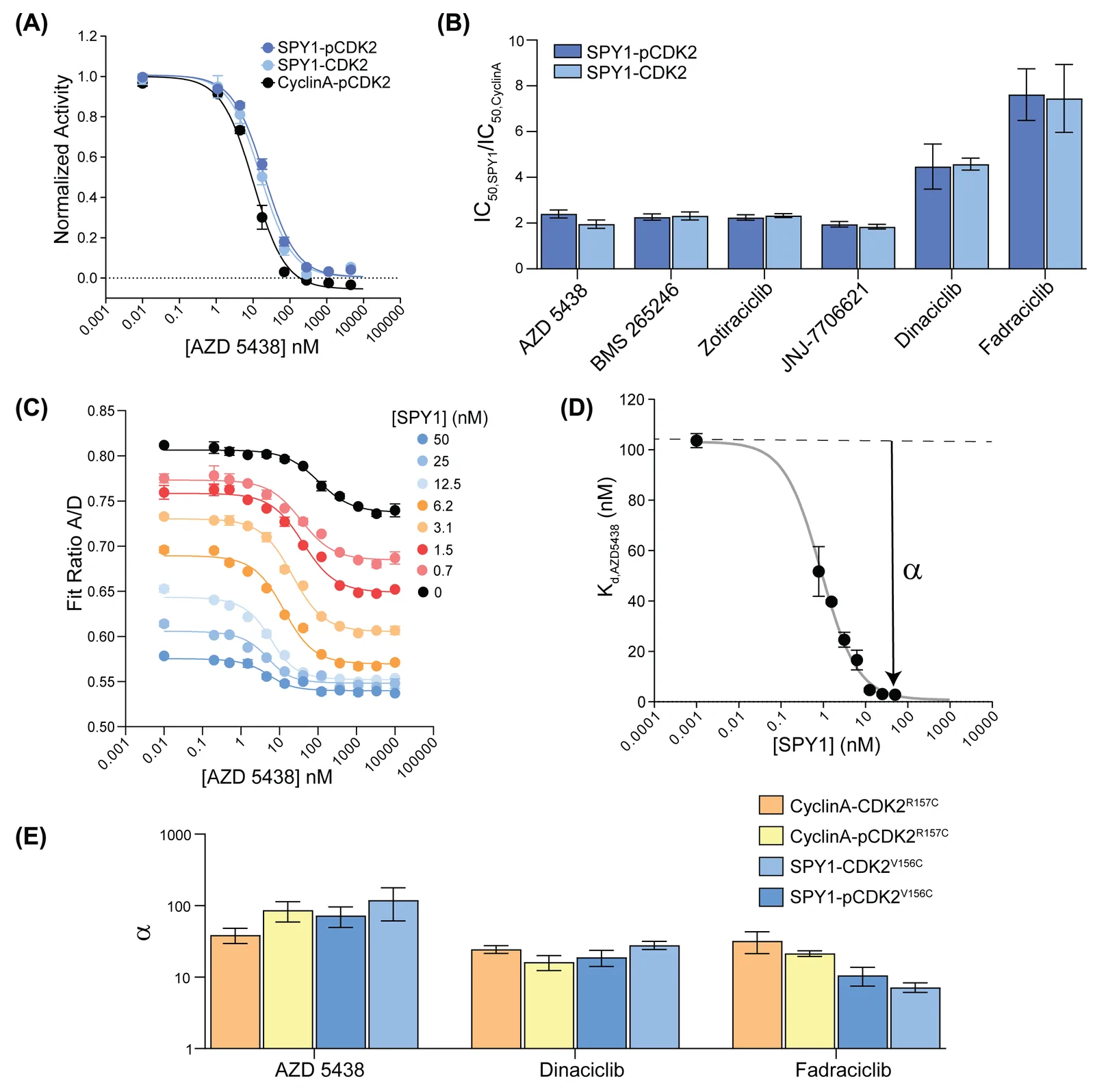
CDK inhibitors show strong positive binding cooperativity with SPY1 and potent inhibition of SPY1–CDK2 complexes (**A**) Representative activity assay comparing inhibition of SPY1–pCDK2 (dark blue) and SPY1–CDK2 (light blue) to CyclinA–pCDK2 (black). Data were normalized to the maximal activity of each complex. (**B**) The ratios of the IC_50_ values for SPY1–pCDK2 and CyclinA–pCDK2 or SPY1–CDK2 and CyclinA–pCDK2 (IC_50,SPY1_/IC_50,CyclinA_) are shown for several CDK inhibitors. Activity data for all inhibitors are shown in Supplementary Figure S2A. (**C**) Representative FRET measurements for AZD5438 binding to pCDK2 at different SPY1 concentrations using the CDK2^V156C^ FRET sensor. Curves represent fits to a quadratic 1:1 binding model (see Methods). FRET data for all inhibitors are shown in Supplementary Figure S3. (**D**) Apparent K_d_ values for AZD 5438, derived from fitting the data in panel (C), plotted as a function of the SPY1 concentration. Data were fit to the quadratic 1:1 binding model (see Methods). The arrow represents the cooperativity value (α) that describes the fold change in affinity of the inhibitor due to the presence of SPY1. (**E**) Plot of cooperativity values (α) for three inhibitors. All plotted data represent mean ± SEM, *n* = 3 independent experiments.

There are limited data available on how ATP-competitive CDK inhibitors interact with SPY1–CDK2. We measured the inhibition of CyclinA–CDK2 and SPY1–CDK2 complexes by the ATP-competitive inhibitor AZD 5438, using the same kinase activity assays described above (Figure 2A). These data showed that the IC_50_ values of AZD 5438 were similar between the complexes, albeit inhibition was slightly weaker for the SPY1–CDK2 complexes. A similar trend in potencies was observed for a panel of ATP-competitive inhibitors (Figure 2B and Supplementary Figure S2), with most inhibitors showing approximately two-fold weaker inhibition of SPY1–CDK2 complexes than CyclinA–pCDK2, although dinaciclib and fadraciclib, which share similar chemical structures, showed larger differences of four- and eight-fold weaker inhibition, respectively.

Most CDK2 inhibitors bind more tightly to activated cyclin–CDK complexes than to monomeric CDK2 [28]. We previously reported that this can be explained by strong allosteric cooperativity between the cyclin subunit and inhibitor binding to CDK2, which arises because cyclin and inhibitor both trigger conformational shifts towards the A-loop_out_ state [27]. Using similar FRET-based experiments as described above, we examined the cooperativity between SPY1 and inhibitor binding to CDK2. These experiments track changes in the A/D ratio as a function of both inhibitor concentration and SPY1/CyclinA concentrations. As observed previously with CyclinA [27], titrations of SPY1 and CDK inhibitors caused coupled reductions in the A/D ratio, reflecting induced conformational shifts of CDK2 towards the A-loop_out_ state. All ATP-competitive inhibitors tested showed substantial positive cooperativity with SPY1, as reflected in a leftward shift of the inhibitor binding curves with higher concentrations of SPY1 (Figure 2C and Supplementary Figure S3). We plotted the values of K_d,app_ for each inhibitor as a function of SPY1 concentration to extract the fold change in inhibitor affinity due to SPY1 binding (cooperativity values, Figure 2D). Figure 2E shows these cooperativity values for both the SPY1 and CyclinA complexes, respectively. SPY1 enhances inhibitor binding to CDK2 by 10–50 fold depending on the inhibitor. The cooperativity values measured for each inhibitor with SPY1 are similar to the corresponding values measured for CyclinA. This observation indicates that the underlying allosteric coupling is similar in the SPY1–CDK2 and CyclinA–CDK2 complexes, with both activator proteins triggering a similar magnitude of conformational shift and promoting inhibitor binding to a similar extent. This explains why most of the inhibitors tested display similar potency for the two complexes. The relatively poor inhibition of SPY1–CDK2 complexes by fadraciclib (Figure 2B) can likely be attributed to its weaker cooperativity with SPY1 than CyclinA (Figure 2E). Although the mechanism responsible for this is unclear, it highlights that the different modes of activation do have allosteric consequences for inhibitor recognition in the ATP-binding site.

### Mutating the SPY1 D136 residue that mimics pT160 converts SPY1 to a cyclin-like activator protein

**Figure 3:**
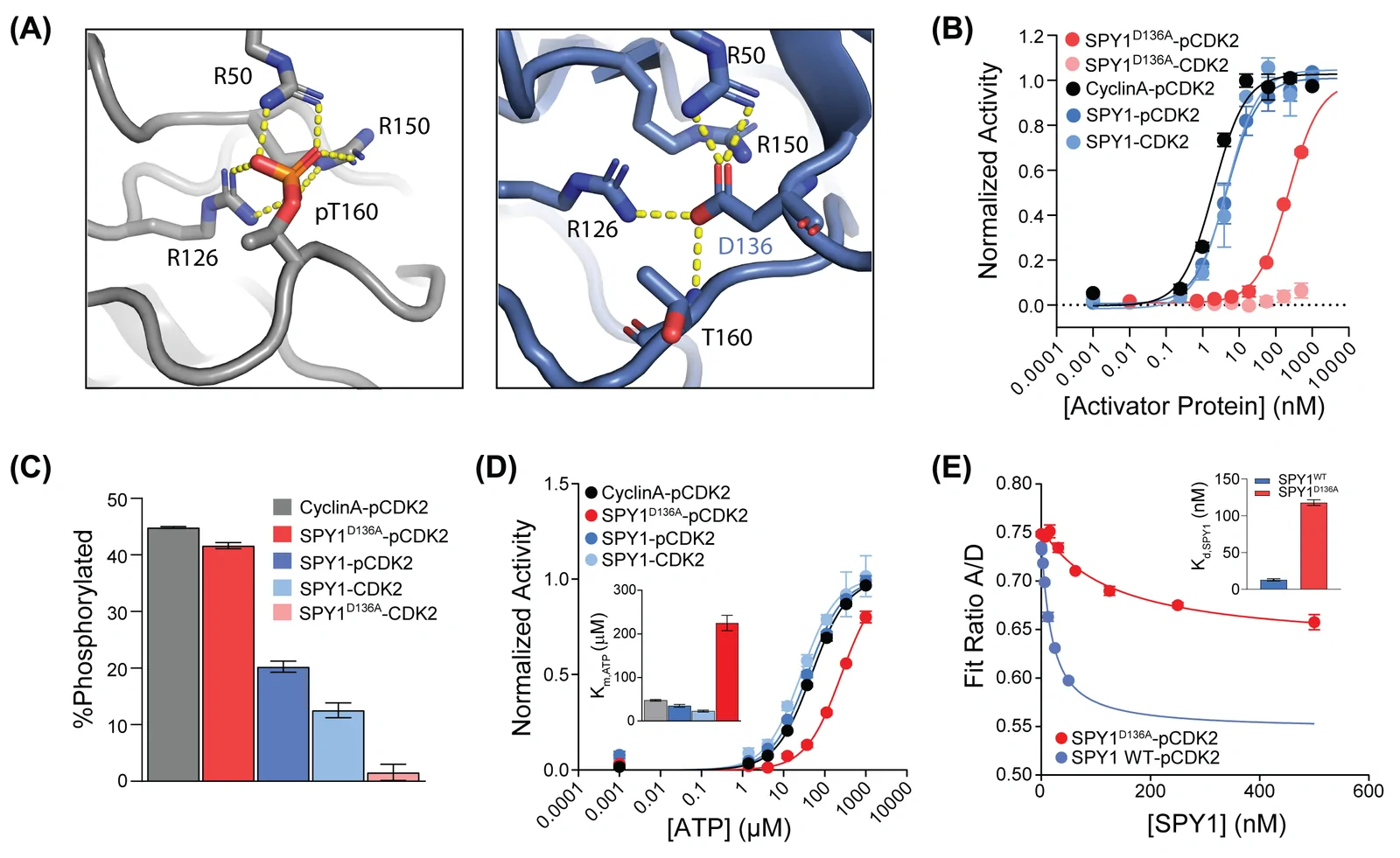
Mutating the SPY1 D136 residue that mimics pT160 converts SPY1 to a cyclin-like activator protein (**A**) X-ray structures of CyclinA–pCDK2 (PDB ID: 1JST) and SPY1–CDK2 (PDB ID: 5UQ2), comparing the electrostatic interactions between the CDK2 arginine residues and either the CDK2 T160 phosphothreonine (left) or the SPY1 D136 residue (right) that stabilize the active state. (**B**) Activity assays comparing SPY1^D136A^–pCDK2 (red) and SPY1^D136A^–CDK2 (pink) to CyclinA–pCDK2 (black), CyclinA–CDK2 (light gray), SPY1^WT^–pCDK2 (dark blue), and SPY1^WT^–CDK2 (light blue), performed at different concentrations of the activator protein. Data were normalized to the maximal activity for each complex. (**C**) Maximal unnormalized activity measured at saturating activator protein for SPY1^D136A^–pCDK2 (red), SPY1^D136A^–CDK2 (pink), CyclinA–pCDK2 (gray), SPY1^WT^–pCDK2 (dark blue), and SPY1^WT^–CDK2 (light blue). Data represent mean ± SEM from a single representative experiment with *n* = 3 replicates. (**D**) Activity assays performed with different ATP concentrations and saturating activator protein concentrations for SPY1^D136A^–pCDK2 (red), CyclinA–pCDK2 (black), SPY1^WT^–pCDK2 (dark blue), and SPY1^WT^–CDK2 (light blue). Data were normalized to the maximal activity for each complex. Inset shows K_m,ATP_ values derived from fitting the data in the main panel. (**E**) FRET data for SPY1^D136A^ (red) or SPY1^WT^ (blue) binding to pCDK2 measured at different concentrations of the activator protein. Curves represent fit to a quadratic 1:1 binding model (see Methods) and the inset shows K_d_ values derived from the fits. Plotted data represent mean ± SEM; *n* = 3 independent experiments.

It is thought that SPY1 can bypass the requirement for CDK2 A-loop phosphorylation due to the position of the SPY1 D136 residue in the SPY1–CDK2 complex, which mimics the T160 phosphothreonine and stabilizes the A-loop_out_ conformation (Figure 3A) [22]. Mutations of SPY1 residues surrounding D136 have been shown to abrogate the proliferative effects of SPY1 *in vivo*, but the role of D136 itself has not been tested [22,29]. We mutated D136 to alanine and tested the ability of the SPY1^D136A^ mutant to activate CDK2 in kinase activity assays. The results confirmed that the SPY1^D136A^ mutant no longer activates unphosphorylated CDK2 (Figure 3B). To our surprise, the results also revealed that the mutant still activates CDK2 phosphorylated on T160, although it exhibits a >10-fold elevated EC_50_ value of 252 ± 30 nM (Figure 3B). The SPY1^D136A^–pCDK2 complex also displays an elevated K_m,ATP_ value compared with SPY1^WT^ (Figure 3D). Nonetheless, when compared at saturating concentrations of the activator proteins and high ATP concentrations, SPY1^D136A^–pCDK2 consistently displayed higher catalytic activity than SPY1^WT^–pCDK2 and approximately equivalent levels of activity to CyclinA–pCDK2 (Figure 3C).

FRET experiments confirmed weaker binding of SPY1^D136A^ to pCDK2 (Figure 3E), with a K_d_ of 125 ± 5 nM for SPY1^D136A^ and 7.8 ± 1.1 nM for SPY1^WT^. Nonetheless, saturating concentrations of SPY1^D136A^ induced substantial reductions in the A/D FRET ratio (albeit smaller than WT SPY1), indicating that despite weaker binding, the mutant still triggers a conformational shift in pCDK2, consistent with its ability to activate phosphorylated CDK2. In contrast, we did not observe a significant reduction in A/D ratio in FRET experiments probing SPY1^D136A^ binding to unphosphorylated CDK2 (Supplementary Figure S4C), indicating that the mutant either does not bind or fails to induce an observable conformational change upon binding. This is consistent with the inability of SPY1^D136A^ to activate unphosphorylated CDK2 in kinase assays. Taken together, these experiments demonstrate that the D136A mutation does not destroy the activating function of SPY1 but instead converts SPY1 to a cyclin-like activator protein that is dependent on T160 phosphorylation for activation of CDK2.

### The D136A mutation restores inhibitor potencies towards SPY1–CDK2 complexes to those observed with CyclinA–pCDK2 complexes

**Figure 4:**
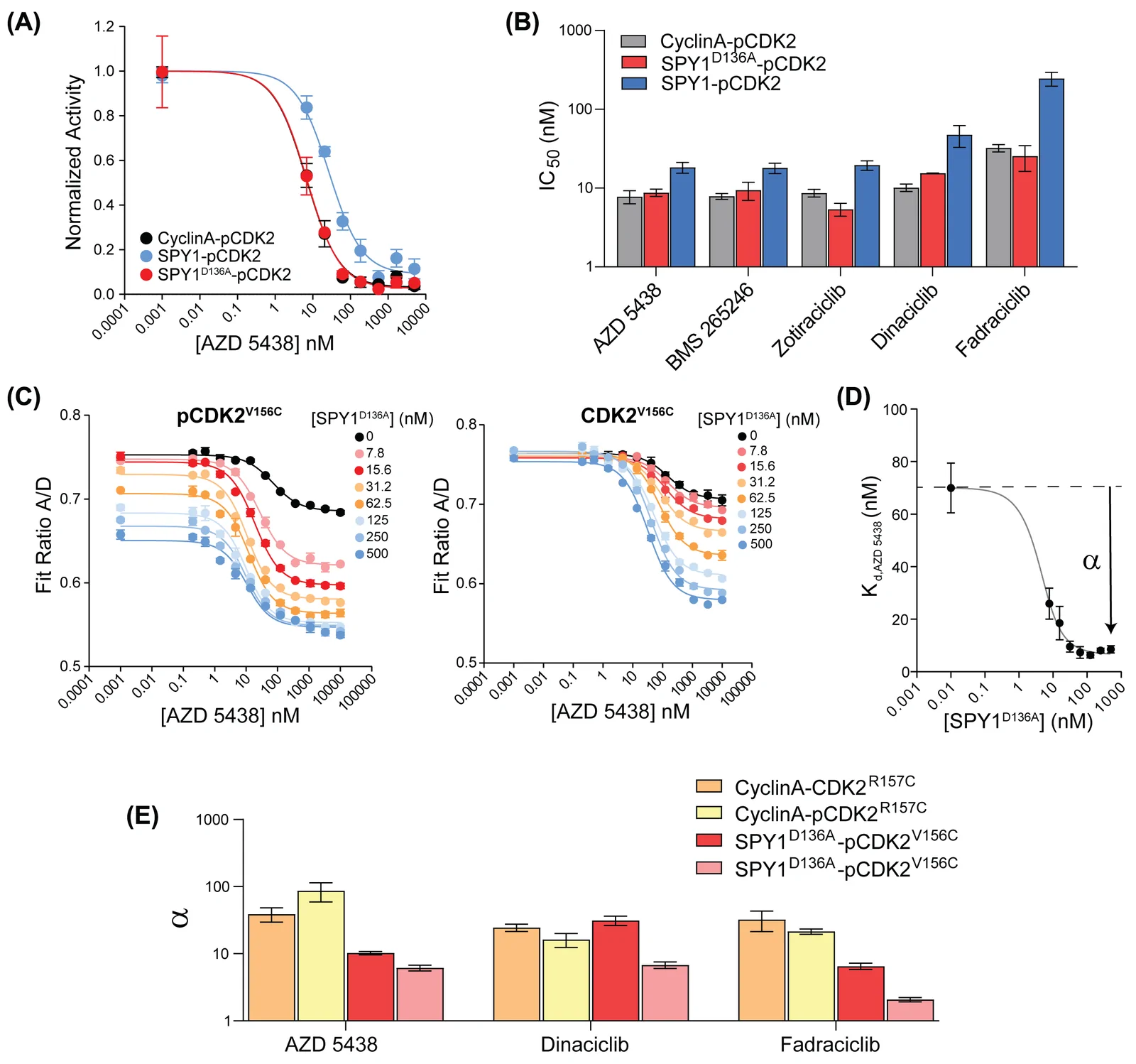
The D136A mutation restores inhibitor potencies for SPY1–CDK2 complexes to those observed with CyclinA–pCDK2 complexes (**A**) Representative activity assays comparing SPY1^D136A^ (red), SPY1^WT^ (blue), and CyclinA (black) with pCDK2 at various concentrations of inhibitor AZD5438. Data were normalized to maximal activity for each complex. (**B**) IC_50_ values for inhibition assays of several CDK inhibitors. Values for AZD5438 are derived from fitting the data in panel (A). Data for all inhibitors are shown in Supplementary Figure S2B. (**C**) Representative FRET measurements for AZD5438 binding to pCDK2 (left) and CDK2 (right) measured with the CDK2^V156C^ FRET sensor at different SPY1 concentrations. Curves represent fits to a quadratic 1:1 binding model (see Methods). Data for all inhibitors are shown in Supplementary Figure S4. (**D**) Apparent K_d_ values for AZD5438, derived from fitting the data in panel (C), plotted as a function of the SPY1^D136A^ concentration. Data were fit to the quadratic 1:1 binding model (see Methods). The arrow represents the cooperativity value (α) that describes the change in affinity of the inhibitor due to the presence of SPY1^D136A^. (**E**) Plot of cooperativity values (α) representing the fold change in inhibitor affinities between monomeric CDK2 and CDK2 bound to either CyclinA or SPY1^D136A^. All plotted data represent mean ± SEM, *n* = 3 independent experiments.

The dampened conformational shift triggered by SPY1^D136A^ binding to pCDK2 observed in the FRET experiments led us to question if inhibitors would still potently inhibit SPY1^D136A^–pCDK2 complex. Inhibition assays showed that the SPY1^D136A^–pCDK2 complex was potently inhibited by AZD 5438, with a similar IC_50_ value to that measured with the CyclinA–pCDK2 complex. Similar results were obtained with several other ATP-competitive inhibitors (Figure 4B), where in each case the IC_50_ values for SPY1^D136A^–pCDK2 were closer to those for CyclinA–pCDK2 than for WT SPY1–pCDK2.

To understand why SPY1^D136A^–pCDK2 remains sensitive to CDK inhibitors, we used FRET experiments to measure the binding cooperativity between SPY1^D136A^ and inhibitors (Figure 4C and Supplementary Figure S4C). Using the pCDK2^V156C^ FRET sensor, we observed coupled reductions in the A/D ratio upon titrating SPY1^D136A^ and inhibitors (Figure 4C, left), similar to those described above for SPY1^WT^. Inhibitor K_d,app_ values extracted by fitting these data (Figure 4D) revealed a similar magnitude of positive binding cooperativity for the SPY1^D136A^–pCDK2 and SPY1^WT^–pCDK2 complexes, demonstrating that the allosteric coupling between inhibitor and activator protein binding is intact in SPY1^D136A^ (Figure 4E and Supplementary Figure S5). These results demonstrate that the D136A mutation does not eliminate the allosteric coupling between SPY1 and inhibitor binding for phosphorylated CDK2.

Parallel experiments performed with the unphosphorylated CDK2^V156C^ FRET sensor showed that, in the absence of inhibitors, no change in FRET is observed even at high SPY1^D136A^ concentrations (Figure 4C, right). However, the response of the unphosphorylated CDK2 FRET sensor to inhibitor binding was enhanced by the presence of SPY1^D136A^ in a dose-dependent manner, indicating simultaneous binding of both inhibitor and activator protein and the presence of some residual cooperativity between the two ligands. Nonetheless, the stronger binding cooperativity observed with pCDK2 is consistent with the large conformational shift triggered by SPY1^D136A^ binding to phosphorylated CDK2 and the lack of a shift upon binding to unphosphorylated CDK2 (Figure 4C). This indicates that for SPY1^D136A^ phosphorylation on T160 is required to help restore allosteric coupling between SPY1 and CDK2.

## Discussion

The requirement for A-loop phosphorylation is a central feature of CDK regulation conserved throughout eukaryotic organisms. SPY1 bypasses this requirement by using a charged residue within its acidic loop that interacts directly with the A-loop, mimicking phosphorylation of T160 and stabilizing the A-loop in the conformation required for substrate binding. SPY1 homologues are present in the most primitive vertebrates [19], and sequence alignments show that the stretch of charged residues containing the D136 residue is broadly conserved, indicating a conserved mechanism for SPY1 activation of CDKs. Utilizing a new stable SPY1 construct, we characterized its interaction with CDK2, confirmed its unusual ability to activate unphosphorylated CDK2, and showed that it is otherwise very similar to CyclinA in terms of CDK2 binding affinity, scale of induced conformational shift, level of catalytic activity of its complexes, and even in the degree to which its binding to CDK2 is allosterically coupled to inhibitor binding. The extent of these similarities is remarkable given that SPY1 shares little if any sequence similarity with the cyclins.

We mutated the D136 aspartic acid residue on SPY1 that acts as a phospho-mimetic residue, expecting that it would abolish all activating potential of SPY1. Instead, the D136A mutation abolished activation of unphosphorylated CDK2, while preserving activation of phosphorylated CDK2 at a similar level to WT SPY1. In other words, the D136A mutation converts SPY1 into a cyclin-like activator protein, albeit one with a weakened binding affinity for CDK2 and an elevated K_m_ value for ATP. An explanation for the retention of the activation function of SPY1^D136A^ towards phosphorylated CDK2 is that when the ionic interactions of the D136 side chain are removed, the T160 phosphothreonine side chain can rotate into its canonical position, rescuing activity by re-establishing the salt-bridging network with the CDK2 arginines and locking the A-loop in the active conformation. The crystal structure of SPY1–CDK2 suggests that this rearrangement can be achieved merely by an 110° rotation of the χ_1_ torsion angle of the phosphothreonine side chain without the need for repositioning the A-loop (Supplementary Figure S6) [22]. Although it is conceivable that such a phosphothreonine rotation model also applies to WT SPY1 when activating pCDK2, our data argue against this. For WT SPY1 interacting with pCDK2 and CDK2, we observed nearly identical binding affinities, K_m,ATP_ values, inhibitor potencies, and magnitudes of induced conformational shift as inferred from the FRET experiments. In contrast, the SPY1^D136A^ mutation reduces binding affinity for pCDK2, increases the K_m,ATP_ value, and decreases the magnitude of conformational shift induced upon binding pCDK2. These observations all point to an important role for the D136 residue in activation of pCDK2 by WT SPY1.

Interestingly, although the SPY1^D136A^–pCDK2 complex is catalytically active, our FRET data suggest that SPY1^D136A^ struggles to fully induce the A-loop_out_ conformation. Because the ionic interactions between D136 and the CDK2 arginine residues only form in the A-loop_out_ state, the mutation might be expected to selectively destabilize the A-loop_out_ state. The elevated K_m,ATP_ value for the mutant is consistent with this model, as nucleotide would have to pay an energetic penalty to bind and induce the A-loop_out_ active state in the mutant. We have previously shown that both nucleotide and substrate peptide binding can induce conformational shifts to the A-loop_out_ state in CDK2 [27].

The WT SPY1–CDK2 complexes displayed somewhat lower overall catalytic activity in our assays compared with the CyclinA–pCDK2 complex. This most likely reflects different preferences of the complexes for the single substrate peptide utilized in our assays. This peptide contains a P + 3 arginine residue that is known to be favored by CyclinA–pCDK2 due to direct interactions between the substrate arginine and the T160 phosphothreonine residue of CDK2 [30]. Previous studies showed that SPY1–CDK2 has a reduced dependence on the P + 3 residue in its substrates [23]. This is consistent with the D136 residue of SPY1 failing to recognize a P + 3 arginine due to its reduced charge and hydrogen bonding capacity compared with phosphothreonine. Interestingly, the SPY1^D136A^ mutant displayed higher maximal activity than WT SPY1 that was similar to CyclinA when tested with phosphorylated CDK2 in the same assay. This is consistent with the hypothesis that the pT160 residue reengages in its canonical active conformation in the SPY1^D136A^–pCDK2 complex, restoring pT160-mediated recognition of the substrate’s P + 3 arginine residue. In cells the different specificities of CyclinA–pCDK2 and SPY1–CDK2 complexes for the residues flanking the phosphoacceptor are likely further modified by differing docking interactions outside the kinase domain. SPY1 does not contain the docking site for a substrate RxL motif [22], a major factor in substrate recognition for the Cyclin–pCDK2 complexes, but instead has been shown to bind to a segment of SUN1 that localizes the SPY1–CDK2 complex to the nuclear membrane [31].

The absence of clear sequence similarity between SPY1 and cyclins has been cited as evidence that they evolved independently and that their activating mechanisms represent an example of convergent evolution [22]. An alternative possibility is that the two lineages share a common ancestor that is obscured by a high degree of sequence divergence. The fact that the D136A mutation converts SPY1 to a cyclin-like activator dependent on T160 phosphorylation could be an indication of a common origin, with the additional ability to activate unphosphorylated CDK2 evolving later.

There has been a resurgence in the clinical development of new CDK2 inhibitors that sidestep off-target inhibition of CDK1, with several new molecules in trials [32,33]. Given the past selectivity challenges, it is interesting to consider whether inhibitors can distinguish between different activation complexes of CDK2. Our experiments show that CDK2 inhibitors bind similarly to and have similar potency for SPY1–CDK2 and CyclinA–CDK2 complexes. This is explained by the fact that SPY1 and CyclinA both exhibit strong binding cooperativity with inhibitors, which boosts inhibitor potency into the low nanomolar range where inhibition is effective. It may seem surprising that structurally distinct binding partners with opposing dependencies on T160 phosphorylation would trigger similar enhancements of inhibitor affinity. However, despite their striking differences, SPY1 and CyclinA induce similar magnitudes of conformational shift towards essentially identical structural states of CDK2. The shared allosteric cooperativity potential of SPY1 and CyclinA is likely due to this conserved conformational shift mechanism. The consequence is similarly potent inhibition of SPY1–CDK2 and CyclinA–CDK2 complexes by small molecules. A contrasting example is provided by the mitotic kinase Aurora A, which can be independently activated by activation loop phosphorylation or by binding of the spindle protein Tpx2 [34]. In this case these regulatory inputs have distinct effects on the conformational landscape of the kinase and differentially affect inhibitor binding [35]. The highly similar conformational responses of CDK2 to SPY1 and CyclinA binding therefore suggest that selective targeting of these complexes with ATP-competitive inhibitors may be challenging. In this light the recently reported alternative allosteric CDK2 inhibitors may be more promising [36].

## Materials and methods

### Expression and purification of CDK2, CyclinA, and SPY1

Human CDK2 (residues 1–298 with a tobacco etch virus (TEV)-cleavable N-terminal hexa-histidine tag in pCDFduet expression vector) were expressed in BL21(DE3) *Escherichia coli* overnight at 18°C. Co-expression with yeast CAK [37] was used to prepare CDK2 phosphorylated on the activation loop (T160) and mass spectrometry was used to confirm single homogenous phosphorylation. Collected cell pellets were resuspended in lysis buffer (50 mM Tris pH 7.5, 500 mM NaCl, 40 mM imidazole, and 10% glycerol) and lysed via sonication (Qsonica). Lysates were clarified by centrifugation and loaded onto pre-equilibrated HisTrap HP IMAC columns (Cytiva), washed with lysis buffer, and eluted in elution buffer (50 mM Tris pH 7.5, 500 mM NaCl, 500 mM imidizole, and 10% glycerol). Imidazole was removed by desalting into lysis buffer, and the histidine tag was cleaved overnight with TEV. TEV-cleaved CDK2 was run over IMAC once again, collecting the pass-through to remove excess TEV, and purified further using a Superdex 75 16/600 HiLoad size-exclusion column (Cytiva).

Bovine CyclinA2 (residues 171–432 with a non-cleavable C-terminal hexa-histidine tag in a pET21D expression vector) was expressed overnight in BL21(DE3) pLysS *E. coli*. Collected cell pellets were lysed as described above, clarified, loaded onto HisTrap HP IMAC columns, eluted off using a linear gradient elution of 0–500 mM imidazole over 20 CV, and stabilized by spiking in MgCl_2_ to a concentration of 100 mM. CyclinA was further purified by size exclusion as described above (50 mM Tris pH 8.25, 100 mM MgCl_2_, 5 mM 2-mercaptoethanol).

Human SPY1 isoform A2 (residues 61–313 with a TEV cleavable N-terminal SUMO solubility tag in a pET28a(+) expression vector) was grown to an O.D. of 0.8 in BL21(DE3) RIL *E. coli* at 37°C. Cultures were then cooled to 18°C and expression was induced by adding IPTG to a final concentration of 0.1 mM. SPY1 was purified as described above for CDK2.

### Kinase activity assays

Activities of the various (p)CDK2 activator protein complexes were determined using the FRET-based Z’-Lyte kinase assay kit (Thermofisher, Ser/Thr 12 peptide). Briefly, protein samples were purified over size exclusion chromatography into Activity Assay Buffer (50 mM Tris pH 7.5, 150 mM NaCl, 10 mM MgCl_2_) supplemented with 0.02% Tween-20 and 0.1 mg/ml of bovine gamma globulin (Sigma). Reactions were run at 5 nM kinase and 500 μM ATP in 384-well plates scanned with a fluorescence plate reader using Magellan Standard software (Tecan) at 25°C. The peptide utilized is related to the CDK7 activation loop sequence containing the phosphorylation sequence S-P-X-R, where X is any amino acid, with a final concentration of 2 μM. Ratiometric FRET values were converted into %phosphorylated according to the Z’-Lyte kit protocol and fit using Prism 10 (GraphPad).

### FRET experiments tracking the A-loop

Appropriate labeling sites were identified by modeling dye conformations using mtsslSuite [38]. “Cys-lite” pCDK2 constructs (C118A, C177S, A93C, and either R157C or V156C for CyclinA [27] and SPY1 experiments, respectively) were labeled with donor (AF488, Fluoroprobes) at a ratio of 0.7:1 for 10 min followed by three-fold excess acceptor (AF568, Fluoroprobes). Labeled samples were concentrated to 500 μl and desalted into Activity Assay Buffer. Desalted samples were then split into two, and one aliquot was treated with lambda phosphate (NEB) to dephosphorylate the A-loop (see Supplementary Figure S1F). Inhibitor dose response titrations were pre-prepared in 384-well plates using the FlexDrop Contactless liquid handler and (p)CDK2 FRET sensors (49 μl) were then dispensed into 384-well plates containing 1 μl of inhibitor in DMSO. Mixtures were shaken for 10 s (Multidrop Combi nL Reagent Dispenser, Thermofisher), centrifuged for 1 min at 1000 rpm, and incubated for 2 h before data collection. After incubation, steady-state fluorescence spectra were collected with a custom-built fluorescence plate reader [39], and the contributions of donor and acceptor fluorescence emission were unmixed by fitting each spectrum to basis functions for AF488, AF568, and the water Raman band as previously described. Titrations of eight concentrations of activator proteins and 12 inhibitor concentrations were used for each inhibitor tested.

### Activity assay and FRET data fitting

Equilibrium dissociation constants (K_d_) of the various inhibitors tested were determined by fitting the fluorescence data to the quadratic 1:1 binding model in GraphPad Prism, which accounts for the significant depletion of ligand arising from high-affinity binding interactions [40]. These K_d_ values measured at different concentrations of activator proteins were fit to the quadratic 1:1 binding model to obtain the fold change in K_d_ value from activator protein binding (cooperativity values). IC_50_ and K_m,ATP_ values were obtained by fitting the processed fluorescence data obtained from the kinase activity assay to either the [inhibitor] versus response (three-parameter) or Michaelis–Menten models in GraphPad Prism, respectively.

## Supplementary Material

Supplementary Figures S1-S6

## Data Availability

All data in the paper are included in the main and supplemental figures. Raw data will be provided upon request.

## Open Access

Open access for this article was enabled by the participation of the University of Minnesota in an all-inclusive Read & Publish agreement with Portland Press and the Biochemical Society under an agreement with the Big Ten Alliance.

## Abbreviations

CAK: CDK-activating kinase
CDKs: cyclin-dependent kinases
TEV: tobacco etch virus
