# Supplementary material for: The non-canonical CDK2 activator SPY1 uses separable mechanisms to activate phosphorylated and unphosphorylated CDK2
Source: Biochem J. 2026 Sep 10;483(10):1917–29. doi: 10.1042/BCJ20260441 (PMC13570426; doi:10.1042/BCJ20260441)
Supplement: Supplementary file 1 [file BCJ-2026-0441_supp.pdf]

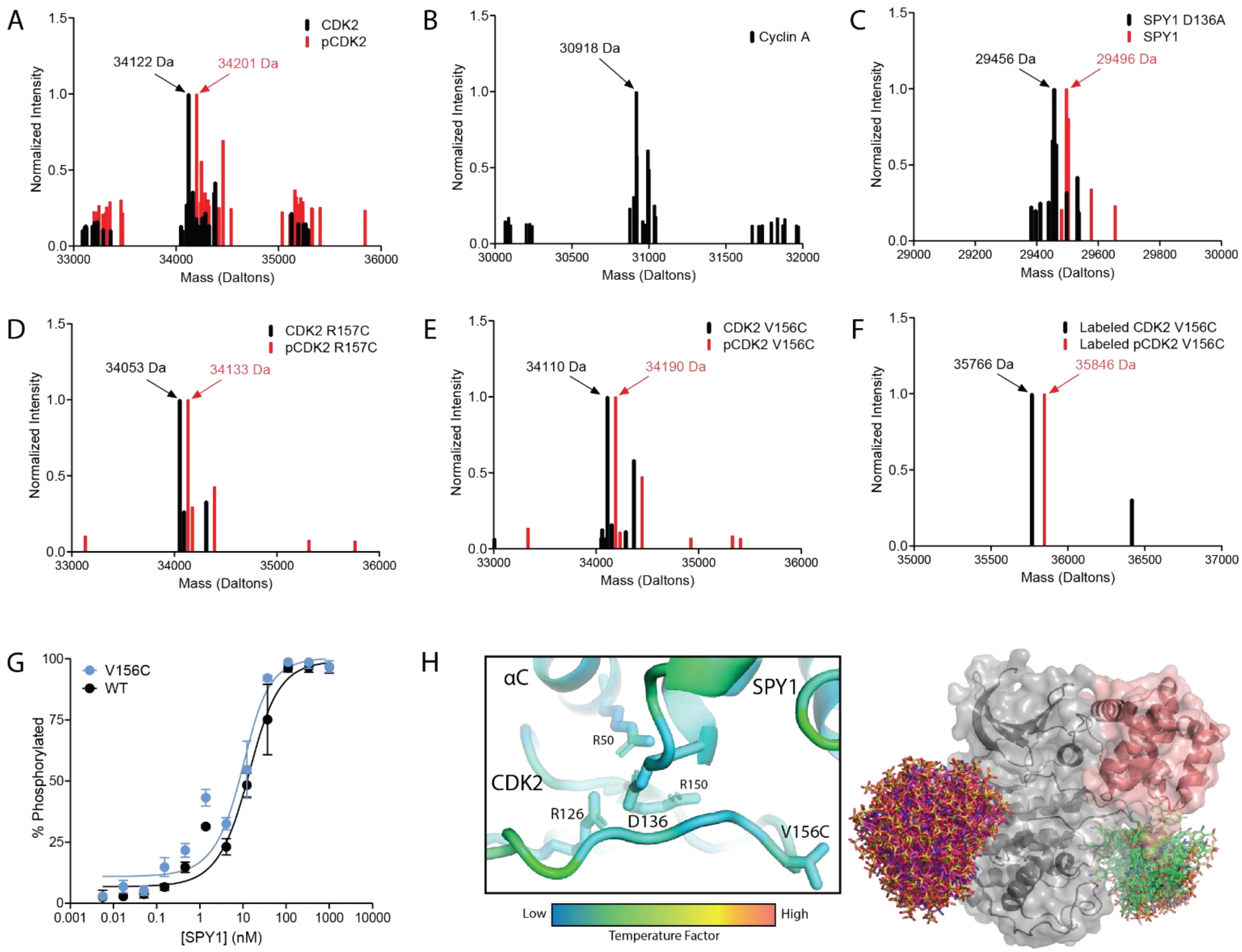

Supplementary Figure 1. Validation of protein constructs, labeling and function| Mass spectra of **A)** WT CDK2 with (red) and without (black) A-loop phosphorylation (+80 Da). Expected mass: 34,204 Da and 34,124 Da respectively. **B)** Bovine CyclinA2. Expected mass: 30,920 Da. **C)** $SPY1^{WT}$ (red) and $SPY1^{D136A}$ (black). Expected mass: 29,503 Da and 29,456 Da respectively. **D)** $CDK2^{A93C,R157C}$ FRET mutant with (red) and without (black) A-loop phosphorylation (+80 Da). Expected mass: 34,052 Da and 34,132 Da respectively. **E)** $CDK2^{A93C,V156C}$ FRET mutant with (red) and without (black) A-loop phosphorylation (+80 Da). Expected mass: 34,112 Da and 34,192 Da respectively. **F)** Doubly-labeled $CDK2^{A93C,V156C}$ FRET mutant (+1,601 Da) with (red) and without (black) A-loop phosphorylation (+80 Da). Expected mass: 35,713 Da and 35,793 Da respectively. **G)** Kinase activity assay showing catalytic activity of the $CDK2^{A93C,V156C}$ FRET mutant (blue) compared to $CDK2^{WT}$ (black) with $SPY1^{WT}$. **H)** Crystallographic temperature factors at the SPY1-CDK2 interface in the vicinity of the V156C labeling site (left) and modeling of C5-Alexa-malemide conformations (right) using MtsslSuite[1] of the A-loop labeling sites for the $CDK2^{A93C,V156C}$ FRET mutant.

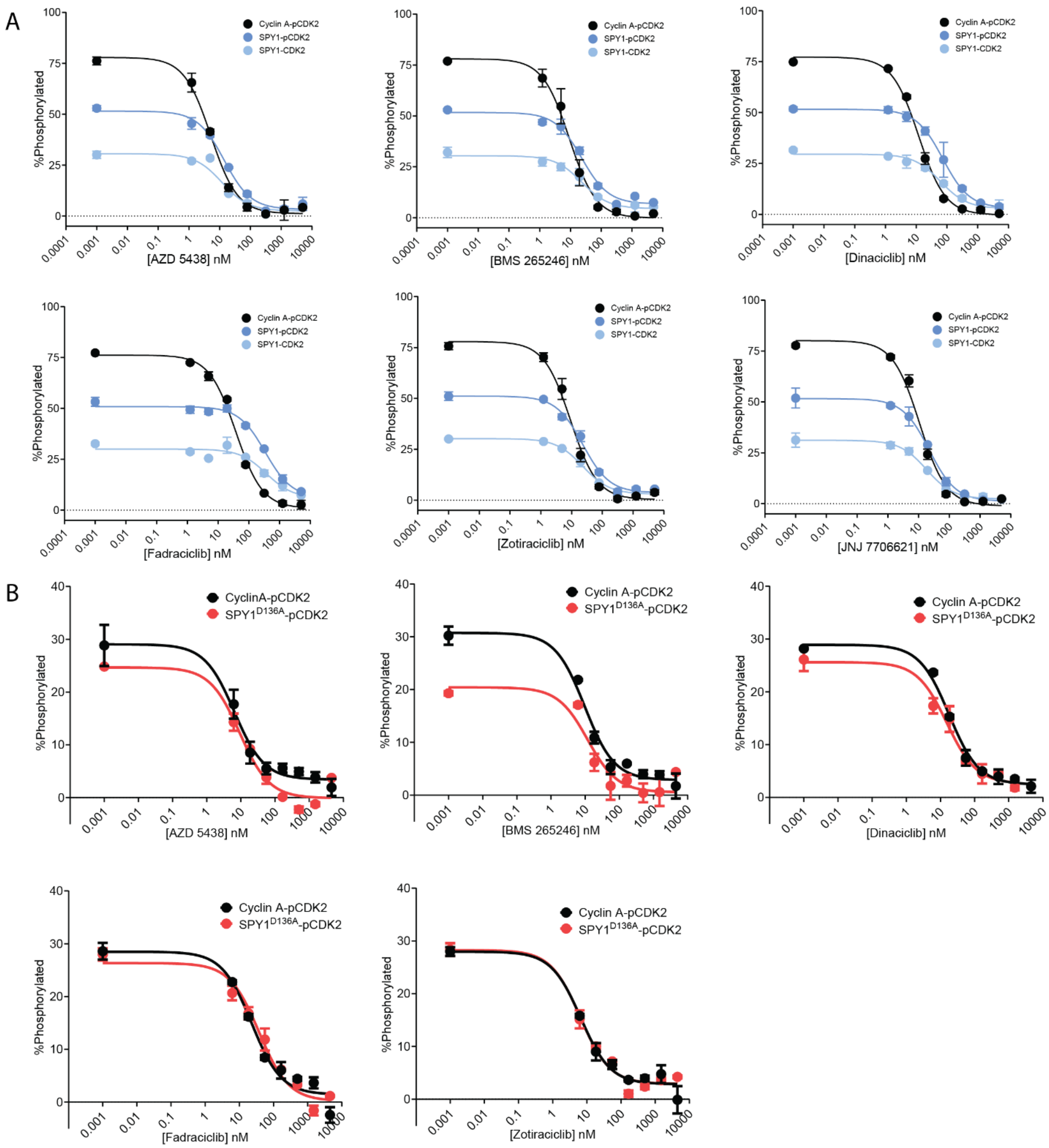

Supplementary Figure 2. ATP competitive inhibition activity assays| Activity assays for CyclinA-pCDK2 (black) and either **A)** SPY1^{WT} with CDK2 (light blue) and pCDK2 (dark blue), or **B)** SPY1^{D136A} with pCDK2 (red).

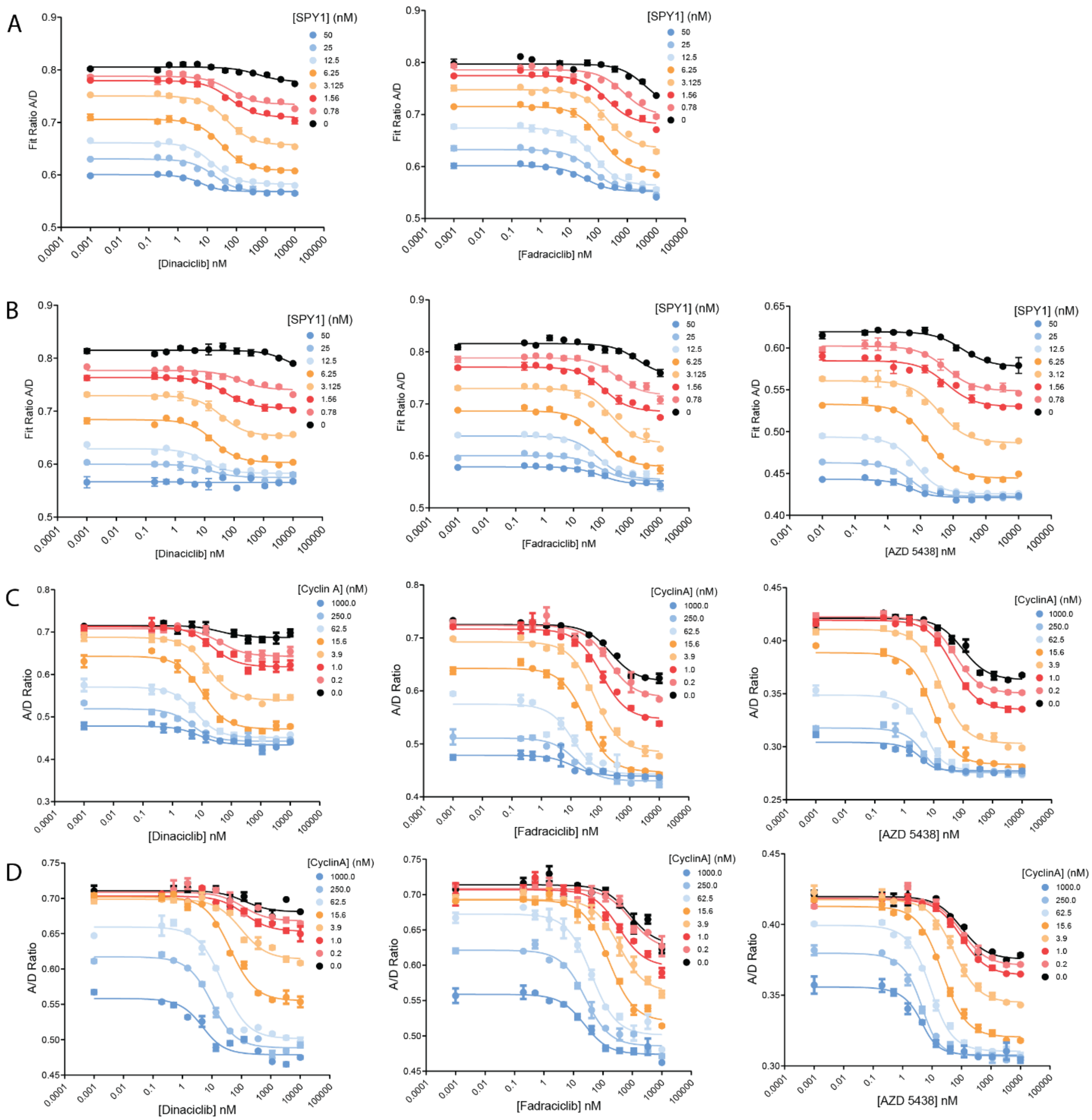

Supplementary Figure 3. FRET measurements of inhibitor binding| Ratiometric FRET measurements and fits are shown for experiments performed with **A)** pCDK2 and SPY1^{WT} with dinaciclib and fadraciclib (AZD5438 is shown in Main Figure 2), **B)** CDK2 and SPY1^{WT}, **C)** pCDK2 and CyclinA, and **D)** CDK2 and CyclinA.

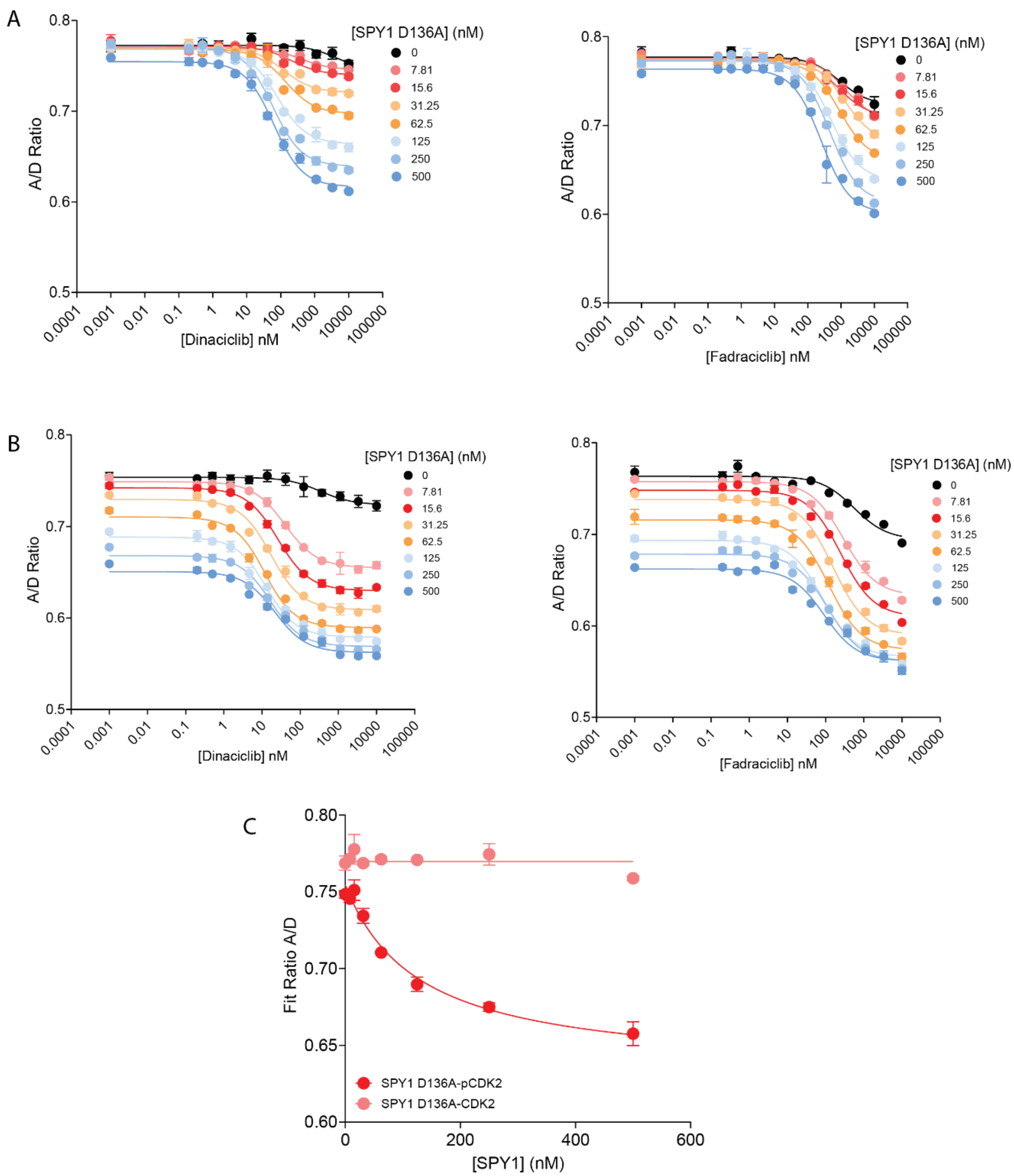

Supplementary Figure 4. FRET measurements of inhibitor binding to SPY1^{D136A}-CDK2 | Ratiometric FRET measurements and fits are shown for experiments performed with SPY1^{D136A} and **A)** CDK2, or **B)** pCDK2. **C)** FRET dose responses for SPY1^{D136A} binding to either CDK2 (pink) or pCDK2 (red) derived from the FRET measurements determined in the absence of inhibitors.

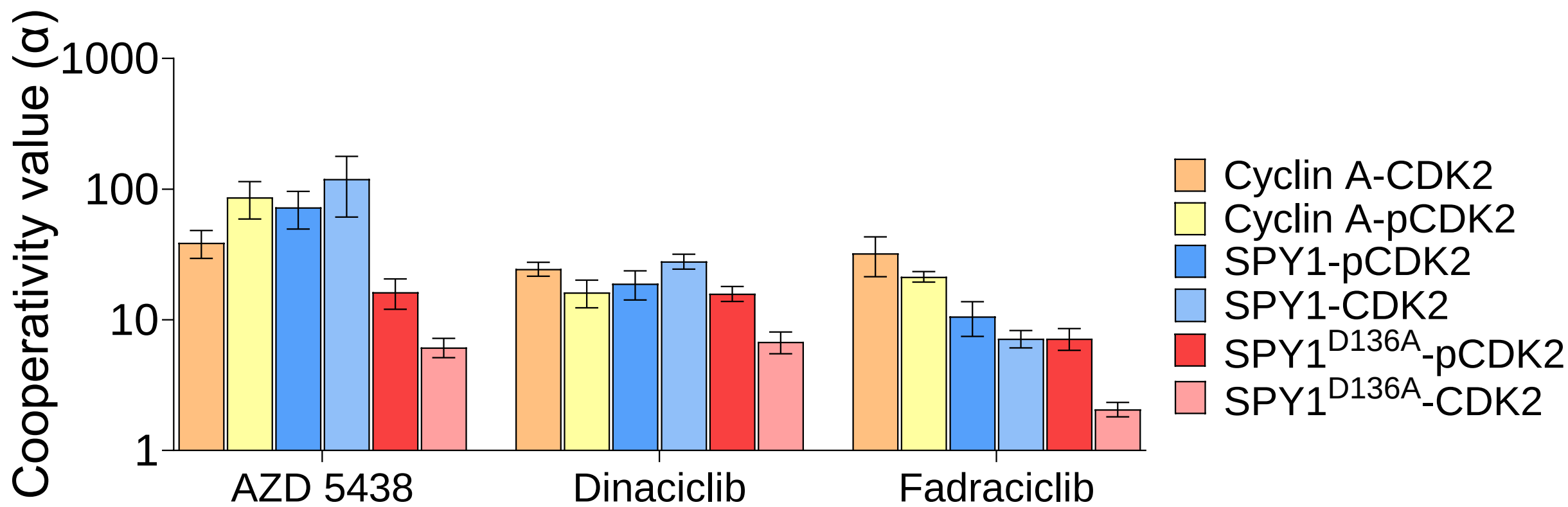

Supplementary Figure 5. Cooperativity values.| Cooperativity values representing changes in drug affinity to CDK2 with and without activator protein bound.

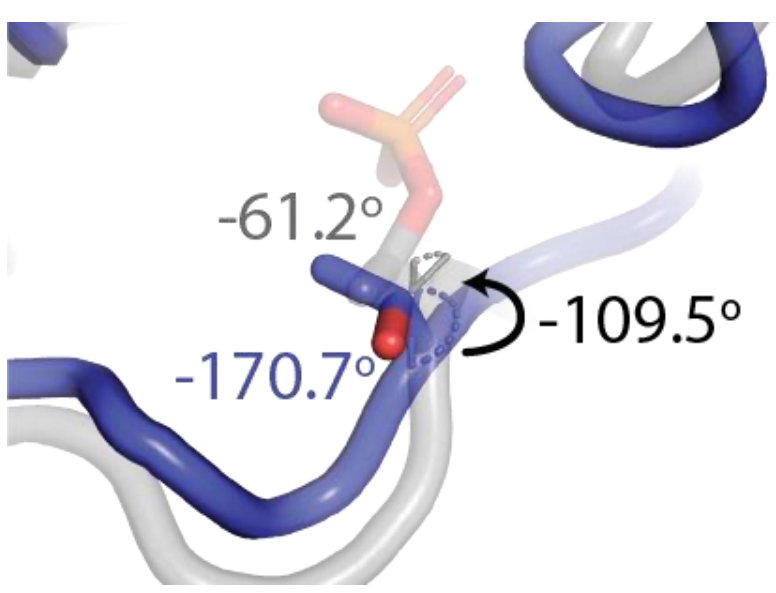

Supplementary Figure 6. χ_1 torsion angle measurements.| Torsion angle measurements for threonine 160 of CDK2 in complex with CyclinA (phosphorylated, grey, PDB:1JST) and SPY1 (blue, PDB: 5UQ2) showing how a phosphothreonine residue in the SPY1^{D136A}-pCDK2 complex would only require a 110° rotation about the χ_1 torsion angle to adopt its canonical position seen in the CyclinA-pCDK2 complex.

References

1. Hagelueken G, Abdullin D, Schiemann O. mtsslSuite: Probing Biomolecular Conformation by Spin-Labeling Studies. Methods Enzymol. 2015;563:595-622. Epub 20150821. doi: 10.1016/bs.mie.2015.06.006. PubMed PMID: 26478500.
